# Case Report: Neoadjuvant FOLFOXIRI followed by laparoscopic en bloc resection and psoas hitch reconstruction for locally advanced colorectal cancer with suspected ureteral invasion: a three-case series

**DOI:** 10.3389/fsurg.2026.1859826

**Published:** 2026-09-21

**Authors:** Daisuke Tomita, Shigeo Toda, Shuichiro Matoba, Yusuke Maeda, Yasutaka Kuno, Jumpei Kashiwagi, Masato Wakamatsu, Yasuhiro Takahashi, Kosuke Hiramatsu, Yutaka Hanaoka, Masashi Ueno, Hiroya Kuroyanagi

**Affiliations:** 1 Department of Gastroenterological Surgery, Toranomon Hospital, Tokyo, Japan; 2 General and Gastroenterological Surgery, Toho University Faculty of Medicine, Tokyo, Japan

**Keywords:** case report, locally advanced colorectal cancer, multivisceral resection, neoadjuvant chemotherapy, psoas hitch procedure

## Abstract

Radical resection is essential for achieving R0 resection and improving prognosis in patients with locally advanced colorectal cancer (CRC) involving adjacent organs. When combined ureteral resection is required, urinary tract reconstruction is critical, as it influences both the risk of complications and postoperative quality of life. Here, we report three cases of locally advanced CRC with suspected ureteral invasion, treated with neoadjuvant chemotherapy (NAC) followed by laparoscopic tumor resection and ureteral reconstruction using the psoas hitch technique, which avoids the use of intestinal segments. Three patients with locally advanced CRC and suspected ureteral involvement received six cycles of FOLFOXIRI-based neoadjuvant chemotherapy (with panitumumab added in one patient). After significant tumor shrinkage, laparoscopic radical resection, including combined ureteral resection, was performed. Ureteroneocystostomy was carried out under direct vision through a lower transverse (Pfannenstiel) incision. Operative times ranged from 505 to 727 min, with no major complications. Postoperative histopathology revealed ypT3N0M0, ypT3N1aM0, and ypT4bN1bM0, respectively, with no pathological evidence of ureteral invasion. All patients received adjuvant chemotherapy and remain recurrence-free to date. Renal function was preserved postoperatively in all cases. We describe three cases in which neoadjuvant chemotherapy effectively shrank locally advanced CRC, yet combined ureteral resection was still necessary to ensure a secure R0 resection. Our approach enabled curative resection while preserving renal function and allowing for the timely initiation of adjuvant chemotherapy. When appropriate cases are selected, the psoas hitch technique can be a feasible and safe method for ureteral reconstruction.

## Introduction

Colorectal cancer (CRC) may invade adjacent organs, including the small intestine, bladder, ureter, and abdominal wall. Achieving an R0 resection is the most important prognostic factor in patients with locally advanced CRC; therefore, extended multivisceral resection (MVR) is often required when adjacent organ invasion is suspected (1). Neoadjuvant chemotherapy (NAC) has recently been introduced as a strategy for locally advanced CRC to downstage tumors, increase the R0 resection rate, and enable evaluation of tumor sensitivity to chemotherapy when planning postoperative treatment (2, 3).

Even when tumor shrinkage is achieved with NAC, it is often necessary to perform combined resection of nearby organs that are suspected to be invaded based on perioperative imaging and intraoperative findings. Particularly in cases of suspected ureteral invasion, a comprehensive treatment strategy that includes ureteral reconstruction is crucial. Ureteroneocystostomy with a psoas hitch is a well-established reconstructive method in urologic practice that avoids the use of intestinal segments and may help preserve renal function and overall treatment tolerance (4, 5).

In this report, we describe three selected cases of locally advanced colorectal cancer in which ureteral invasion was strongly suspected based on intraoperative findings, and in which the tumor–ureter interface at the level of the common iliac artery made ureteroneocystostomy with psoas hitch procedure the most appropriate reconstructive option. Since all three cases achieved R0 resection and are currently free of recurrence without any renal dysfunction, we report on the feasibility and safety of our treatment strategy.

## Case presentation

This case series describes three patients with locally advanced colorectal cancer suspected of ureteral invasion. Since R0 resection was considered challenging, they received six cycles of 5-fluorouracil, folinic acid, oxaliplatin, and irinotecan (FOLFOXIRI)-based neoadjuvant chemotherapy. After confirming tumor shrinkage, they underwent radical resection between March 2021 and January 2023 at our institution. All three patients initially presented with bowel obstruction caused by colorectal cancer and required urgent creation of a diverting stoma. On preoperative computed tomography (CT) prior to emergency surgery, the thickened colonic wall was in contact with the ureter in all cases, and hydronephrosis was observed in two of the three patients. During stoma creation, direct intraoperative inspection revealed dense adhesion or inseparable contact between the tumor and the ureter, raising a strong suspicion of ureteral invasion. Before definitive curative resection, all patients underwent contrast-enhanced CT, pelvic magnetic resonance imaging, and positron emission tomography/CT to evaluate the response to neoadjuvant chemotherapy and reassess the tumor–ureter relationship. Although radiological results showed an improvement in hydronephrosis after NAC, *en bloc* ureteral resection was planned if a safe dissection plane could not be identified during the second surgery to avoid an R1 resection and ureteral devascularization/injury. Formal preoperative urologic assessment was performed in all patients. The colorectal surgical team determined whether ureteral preservation was oncologically feasible, while the extent of ureteral resection and reconstructive strategy were determined in collaboration with the urology team when resection was required. The choice of reconstruction method depends on the length of the resected ureter. For a defect or insufficiency in the lower ureter, we opt for ureteral end-to-end anastomosis for defects of 2–3 cm, the psoas hitch technique for defects of 4–10 cm, and the Boari flap technique, which creates a lumen in the anterior bladder wall, for defects of 11–15 cm. In our cases, due to the tumor-ureter interface at the level of the common iliac artery, ureteroneocystostomy using the psoas hitch technique was considered the most appropriate reconstructive option.

The baseline characteristics are provided in Table 1, and surgical and histopathological findings are shown in Table 2.

**Table 1:** Baseline characteristics of the patients.

Case No	1	2	3
Age, years	57	68	68
Sex	Male	Male	Female
Tumor localization	Sigmoid colon	Rectosigmoid colon	Sigmoid colon
Subjective symptoms	Abdominal distention	Abdominal distention	Diarrhea and abdominal discomfort
Preoperative histological diagnosis	Moderately differentiated Adenocarcinoma	Poorly differentiated Adenocarcinoma	Moderately differentiated Adenocarcinoma
Preoperative CEA, ng/mL (→ ^a^Post neoadjuvant chemotherapy)	1.3	388.3→43.7	42.8→11.0
Preoperative CA19-9, U/mL (→ ^a^Post neoadjuvant chemotherapy)	5	1,547→1,304	12
MMR/MSI status	pMMR	pMMR	pMMR
RAS mutation	Wild type	Mutant	Mutant
BRAF mutation	Wild type	Wild type	Wild type
Preoperative serum Cre level, mg/dL	0.69	0.73	0.56
Neoadjuvant chemotherapy	FOLFOXIRI + Pmab	FOLFOXIRI	FOLFOXIRI
Neoadjuvant radiotherapy	None	Short-course radiotherapy (25.0 Gy in 5 days)	None
Clinical Stage (UICC 8th)	cT4b(right ureter)N2bM0 →ycT4bN0M0	cT4b(left ureter and left seminal vesicle)N2aM0 →ycT4bN2aM0	cT4b(right ovary, uterus, and right ureter)N0M0 →ycT4bN0M0

a Post-treatment values are shown for patients with elevated preoperative levels. *CEA*, carcinoembryonic antigen; *CA19-9*, carbohydrate antigen 19-9; *MMR/MSI*, mismatch repair/microsatellite instability; *pMMR*, proficient MMR; *RAS*, Rat sarcoma; *BRAF*, B-Raf proto-oncogene, serine/threonine kinase; *Cre*, creatinine; *FOLFOXIRI*, 5-fluorouracil + folinic acid + oxaliplatin + irinotecan; *Pmab*, panitumumab.

**Table 2:** Surgical and histopathological findings.

Case No	1	2	3
Operative method	Sigmoid colostomy closure, laparoscopic low anterior resection, partial combined resection of appendix, right spermatic duct and right ureter, ureteroneocystostomy with psoas hitch procedure, temporary ileostomy	Laparoscopic Hartmann's procedure, left lateral lymph node dissection, combined resection of left ureter and left seminal vesicle, ureteroneocystostomy with psoas hitch procedure	Sigmoid colostomy closure, laparoscopic high anterior resection, combined resection of right ovary, uterus, appendix, and partial resection of right ureter, ureteroneocystostomy with psoas hitch procedure, temporary ileostomy
Operative time	525 min	727 min	505 min
Blood loss	0 mL	300 mL	560 mL
Postoperative complications	None	Ileus (Clavien-Dindo classification grade II)	None
Postoperative hospital stay	11 days	19 days	14 days
Postoperative urinary catheter removal	7 days	10 days	7 days
Postoperative ureteral stent removal	7 days	7 days	7 days
Tumor size^a^	38 × 14 mm	55 × 14 mm	100 × 70 mm
Macroscopic classification	Type 2	Type 5	Type 2
Pathological feature	Moderately differentiated Adenocarcinoma	Moderately and poorly differentiated Adenocarcinoma	Moderately differentiated and mucinous Adenocarcinoma
Pathological Stage (UICC 8th)	ypT3N0 (0/55) M0	ypT3N1a (1/19) M0	ypT4b (appendix and right ovary) N1b (2/20) M0
Venous invasion	Negative	Negative	Positive
Lymphatic invasion	Negative	Negative	Negative
Resection margin	Negative	Negative	Negative
Circumferential resection margin	15 mm	2 mm	4 mm
Post-operative CEA, ng/mL	2.1	8.8	1.5
Post-operative CA19-9, U/mL	8	95	7
Adjuvant chemotherapy	CAPOX	FOLFOX	CAPOX
Recurrence free survival	60 months	49 months	36 months
Serum Cre level at 6 months post-surgery, mg/dL	0.65	0.75	0.63
Cre at 1 year, mg/dL	0.72	0.70	0.75
Cre at 2 years, mg/dL	0.82	0.99	0.86
Cre at final follow-up, mg/dL	0.77	0.74	0.76

a (Circumference diameter) × (Lesion length). *CEA*, carcinoembryonic antigen; *CA19-9*, carbohydrate antigen 19-9; *CAPOX*, capecitabine + oxaliplatin; *FOLFOX*, folinic acid, fluorouracil, and oxaliplatin; *Cre*, creatinine.

### Case 1

A 57-year-old man visited his previous physician, primarily complaining of abdominal distention. He was referred to our hospital after a colonoscopy revealed a tumor in the sigmoid colon that was causing an obstruction. A CT scan showed that the patient had sigmoid colon cancer with abscess formation and multiple enlarged pericolic lymph nodes (Figures 1A,B). Right hydronephrosis was observed; thus, tumor invasion of the right ureter was also suspected. There was no evidence of distant metastasis. Because there was a concern that the resection margin would be positive if upfront surgery were performed, the patient received six courses of FOLFOXIRI + Panitumumab (Pmab) chemotherapy after constructing the sigmoid colostomy. NAC demonstrated significant tumor shrinkage (Figures 1C,D), leading to the decision to proceed with resection of the primary tumor. Intraoperative findings suggested involvement of the appendix, right spermatic duct, and right ureter, which were therefore resected *en bloc* with the tumor. The urinary tract was reconstructed using the psoas hitch technique, and a temporary ileostomy was created due to the lower-than-expected placement of the intestinal anastomosis. The surgery lasted 525 min with minimal blood loss. The patient was discharged from the hospital 11 days after the surgery. Postoperative pathology revealed ypT3N0M0. There was no renal insufficiency, and the patient received four cycles of capecitabine and oxaliplatin (CAPOX) as adjuvant chemotherapy. The temporary ileostomy was closed six months later, and the patient remained recurrence-free for 60 months.

**Figure 1:**
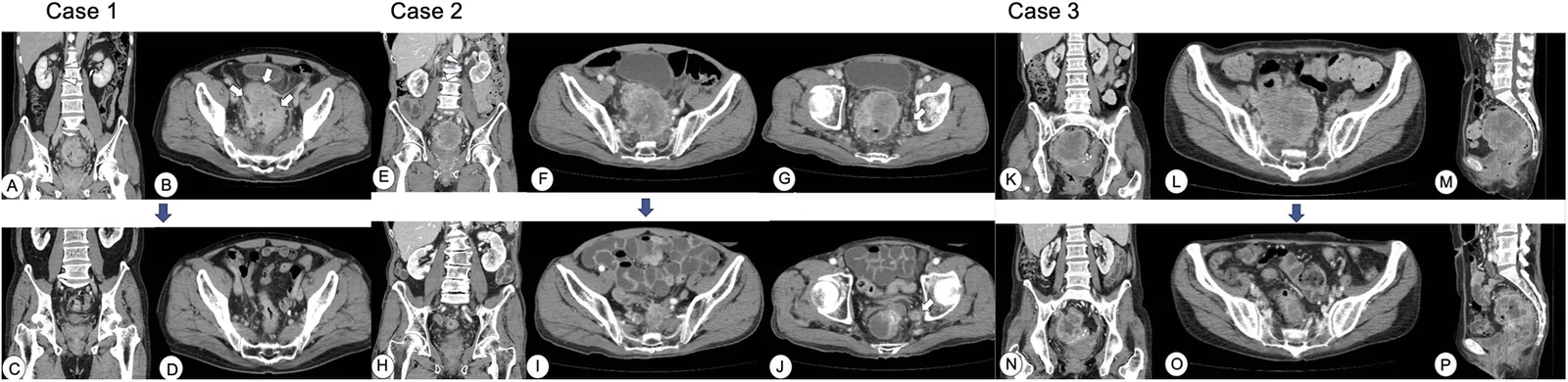
CT findings of case 1 **(A)-(D)** The coronal **(A)** and axial CT **(B)** before NAC. Sigmoid colon cancer had invaded the right pelvic wall and abscess formation (white arrow) was observed. Right hydronephrosis (white arrowhead) was also observed. The coronal **(C)** and axial CT **(D)** after NAC. The tumor was markedly reduced. Abscess formation had also shrunk. CT findings of Case 2 **(E)-(J)** The coronal **(E)** and axial CT **(F,G)** before NAC. Rectosigmoid colon cancer had invaded the pelvic wall. Left hydronephrosis (white arrowhead) was also observed. The left lateral lymph node was enlarged (white arrow). The coronal **(H)** and axial CT **(I,J)** after NAC. The tumor was markedly reduced. The left lateral lymph node remained enlarged (white arrows). CT findings of Case 3 **(K)-(P)** The coronal **(K)**, axial **(L)** and sagittal CT **(M)** before NAC. Sigmoid colon tumor had invaded the right ovary and uterus. Right ureteral invasion was also suspected. The coronal **(N)**, axial **(O)** and sagittal CT **(P)** after NAC. Tumor size was reduced, but invasion of surrounding organs was still suspected.

### Case 2

A 68-year-old man presented to our department with distention in the lower abdomen. A CT scan revealed a rectosigmoid lesion that had invaded the pelvic wall, and an enlarged left lateral lymph node was found, suggestive of lymph node metastasis (Figures 1E–G). Additionally, infiltration of the left ureter was suspected due to the presence of left hydronephrosis. After a colonoscopic biopsy confirmed a diagnosis of adenocarcinoma, a sigmoid colostomy was performed. Following six courses of FOLFOXIRI chemotherapy, the tumor shrank and became resectable (Figures 1H–J). Given the presence of lateral lymph node metastasis and proximity to the pelvic sidewall, short-course radiotherapy (total dose of 25.0 Gy in 5 days) was added according to our institutional protocol. During the laparoscopic curative resection, the sigmoid colostomy was not reversed because it did not interfere with the intraoperative procedures. The surgical approach included a laparoscopic Hartmann's procedure, along with resection of the left ureter, left seminal vesicle, and left lateral lymph node dissection. Due to significant tumor invasion into the pelvic wall, partial resection of the left and right pelvic plexus was also necessary. The urinary tract was reconstructed using the psoas hitch technique. The operation lasted 727 min and involved blood loss of 300 mL. On the second postoperative day, the patient developed ileus (Clavien-Dindo classification grade II). Nevertheless, he experienced no major complications and was discharged on the 19th postoperative day. Postoperative pathology indicated ypT3N1a (1/19) M0, with the lateral lymph node testing positive for metastasis. The patient subsequently received six cycles of adjuvant chemotherapy with folinic acid, fluorouracil, and oxaliplatin (FOLFOX). The patient remains recurrence-free 49 months after surgery.

### Case 3

A 68-year-old woman presented to our department with diarrhea and abdominal discomfort. CT showed a sigmoid colon tumor invading the right ovary and uterus (Figures 1K–M). Right hydronephrosis was not observed, but right ureteral invasion was suspected based on the location of the tumor. After constructing a colostomy of the sigmoid colon, NAC was introduced. After six courses of FOLFOXIRI, the tumor had shrunk and was considered to be resectable (Figures 1N–P); laparoscopic resection was therefore performed. The sigmoid colostomy was closed first, and then the patient underwent a laparoscopic procedure. Intraoperative findings suggested involvement of the appendix, right ovary, uterus, and right ureter, which were therefore resected *en bloc* with the tumor. The urinary tract was reconstructed using the psoas hitch technique, and finally, an ileostomy was created. The operation time was 505 min with a blood loss of 560 mL, and a blood transfusion was required. The patient had no postoperative complications and was discharged from the hospital on the 14th postoperative day. Postoperative pathological findings were ypT4bN1b (2/20) M0, with tumor invasion into the appendix and right ovary. The patient received four cycles of CAPOX as postoperative adjuvant chemotherapy, and closure of the temporary stoma was performed four months after colon resection. She is alive 36 months postoperatively without recurrence.

### Operation procedure

After administering general anesthesia, the patient was positioned in the lithotomy position. In Cases 1 and 3, the sigmoid colostomy closure was performed first. Utilizing the open technique, a 12 mm trocar was inserted through a transumbilical incision. The abdomen was then insufflated with carbon dioxide to a pressure of 8 mmHg. An intraperitoneal observation was conducted to confirm the absence of obvious liver metastasis or peritoneal dissemination. Subsequently, 5 mm trocars were inserted in the right upper abdomen, left upper abdomen, and left lower abdomen, while a 12 mm port was placed in the right lower abdomen. Organs that were suspected to have tumor invasion were resected along with the tumor in each case. Ligation of the inferior mesenteric artery root and D3 lymph node dissection were performed in all cases. Additionally, a left lateral lymph node dissection was carried out in Case 2.

### The psoas hitch procedure

After confirming that safe dissection between the tumor and the ureter was not feasible (Figure 2A), the ureter was dissected at the level of the common iliac artery. The distal ureteral stump on the bladder side was closed with a clip. Laparoscopic bladder mobilization was performed to facilitate subsequent ureterovesical anastomosis. This involved meticulous dissection of the bladder from the anterior and lateral abdominal walls to ensure optimal mobility (Figure 2B). The peritoneum lateral to the medial umbilical ligament was incised, and the space of Retzius was accessed and dissected down to the pubic bone. A transverse (Pfannenstiel) incision was made in the lower abdomen on the side of ureteral resection (right in Cases 1 and 3; left in Case 2). Using this transverse incision, we performed a ureterovesical anastomosis under direct vision. After bladder wall incision, the ipsilateral superior aspect of the bladder was assessed for reach to the intended psoas muscle fixation point (Figure 2C). For fixation of the bladder at the psoas muscle, 3/0 monofilament absorbable sutures were placed through the psoas tendon above the common iliac artery, taking care to avoid injury to the femoral branch of the genitofemoral nerve (Figure 2D). The bladder mucosa was incised at the anticipated ureteral orifice, and a 3 cm submucosal tunnel was created. The ureter was then advanced into this tunnel, ensuring no kinking or obstruction (Figure 2E). Subsequently, the psoas fixation sutures were tied. Ureteral reimplantation was accomplished with six 5/0 monofilament absorbable sutures encompassing the ureteral wall and bladder mucosa at the orifice (Figure 2F). Following completion of the anastomosis, a 7Fr double-J ureteral stent was inserted, confirming unimpeded placement. The bladder was closed in two layers: the entire bladder layer with a 3/0 monofilament absorbable running suture, followed by detrusor closure with 3/0 monofilament absorbable sutures (Figure 2G). After completing the ureteral reimplantation under direct vision, we returned to the laparoscopic view to reconfirm that bladder mobilization was adequate and that the reconstructed ureter was tension-free (Figure 2H). Specifically, we checked for ureteral twisting and ensured the angle of the submucosal tunnel was smooth and not excessively acute. The urinary catheter was removed 7–10 days postoperatively, and the ureteral stent was extracted seven days after surgery.

**Figure 2:**
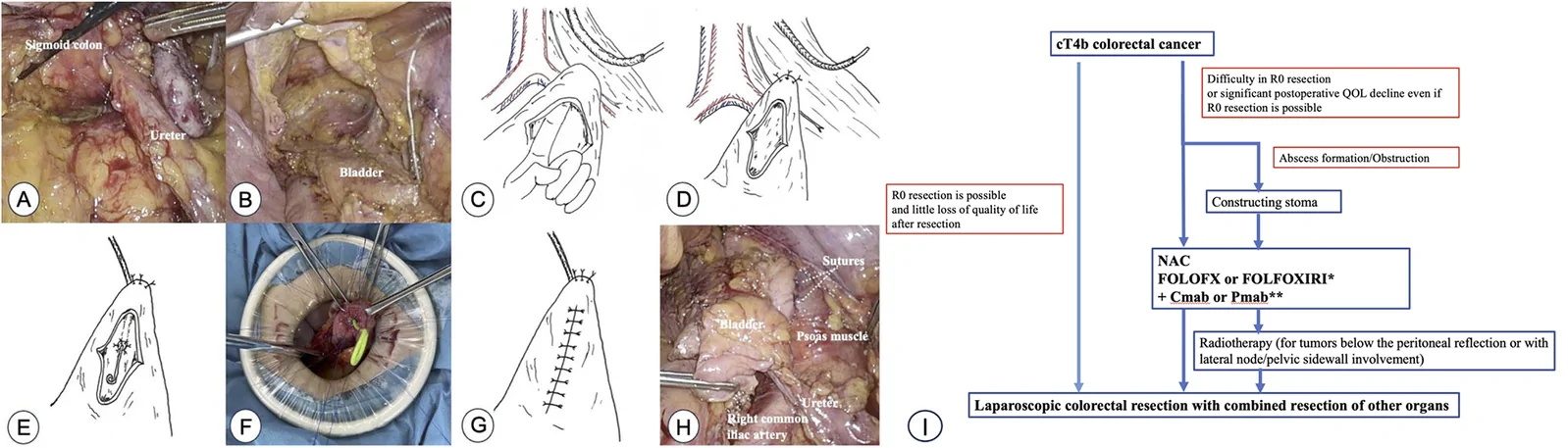
Intraoperative images and schema of the psoas hitch procedure **(A)-(H) (A)** The suspected ureteral invasion before resection. **(B)** Both the prevesical and lateral bladder spaces were widely mobilized as much as possible. **(C)** The surgeon's finger opened and guided the bladder wall to the psoas muscle. **(D)** Three sutures were placed through the psoas muscle and the bladder. **(E)** The ureter was reimplanted with a submucosal tunnel and anastomosed to the bladder wall. The Double-J ureteral stent was then placed. **(F)** Intraoperative image of ureteroneocystostomy. **(G)** The bladder wall was closed in two layers. **(H)** We reconfirmed that the reconstructed ureter was tension-free in the laparoscopic view. **(I)** Treatment strategy for cT4b left side colorectal cancer in our department. Cmab, cetuximab; FOLFOX, folinic acid, fluorouracil, and oxaliplatin; FOLFOXIRI, 5-fluorouracil + folinic acid + oxaliplatin + irinotecan; NAC, neoadjuvant chemotherapy; Pmab, panitumumab; QOL, quality of life. *In cases with RAS mutant type. **In cases with RAS wild type.

## Discussion

In CRC invading adjacent organs, achieving R0 resection through *en bloc* resection is critically important, as it has been reported to be a key factor for long-term survival (1). While concerns have been raised regarding the potential for overtreatment and delays in subsequent tumor management, several studies have confirmed the feasibility and safety of NAC in patients with locally advanced CRC (2, 3). The treatment strategy for locally advanced colorectal cancer (cT4) in our department is outlined in Figure 2I. If R0 resection is feasible and the likelihood of a substantial decline in quality of life is low, upfront surgery is performed. If achieving R0 resection is considered challenging, or if extensive MVR with substantial functional impairment is anticipated, preoperative chemotherapy is administered. The systemic regimen is individualized according to tumor characteristics, including molecular status, and includes FOLFOX or FOLFOXIRI, with the addition of panitumumab or cetuximab for patients with RAS wild-type tumors. Bevacizumab is generally avoided because of concerns regarding perioperative complications such as hemorrhage, delayed wound healing, and anastomotic leakage. Radiation therapy is considered selectively when the risk of local recurrence is high, such as tumors extending below the peritoneal reflection or with lateral lymph node metastasis or pelvic sidewall involvement.

The optimal duration of NAC and the appropriate interval between chemotherapy and surgery for locally advanced CRC have not been established. At our institution, tumor response is assessed after four cycles of NAC, and when tumor shrinkage is achieved without disease progression or unacceptable toxicity, two additional cycles are administered. Definitive surgery is generally performed approximately 2–4 weeks after the final cycle, after confirming adequate recovery from treatment-related toxicity. Thus, both the duration of NAC and the timing of surgery reflect our institutional practice rather than established standards.

Because organ adherence does not always reflect true invasion in clinical T4b disease, discordance between cT4b and pT4b is common. For example, Stief et al. confirmed pT4b invasion in only 22% (16/72) of patients undergoing combined ureteral resection (6). Nonetheless, even after tumor regression with NAC, *en bloc* resection of adherent organs remains indicated, as current oncologic guidelines prioritize avoiding R1 resection and recommend no-touch *en bloc* resection whenever malignancy cannot be definitively excluded (7).

In our practice, the indication for ureteral resection is determined by combining findings before and after NAC with intraoperative assessment. When pre-NAC imaging and initial exploration indicate dense tumor–ureter adhesion, potential invasion is assumed despite radiographic response; forced dissection should be avoided to prevent tumor exposure, warranting *en bloc* resection. In all three of our patients, dense adhesion observed during initial emergency surgery persisted at the time of definitive resection. Although frozen section analysis was available, dissection between the tumor and ureter was considered hazardous, as post-NAC inflammatory fibrosis often mimics tumor invasion and attempts at preservation risk R1 margins, ureteral devascularization, or perforation. Therefore, ureter-preserving dissection was not attempted. All patients achieved negative margins and remain free of local recurrence, which is consistent with the oncologic adequacy of this strategy.

Laparoscopic MVR for clinically suspected T4 colorectal cancer has been reported to achieve R0 rates, recurrence, and survival comparable to those of open surgery in appropriately selected patients (8, 9). In the present series, the laparoscopic approach facilitated bladder mobilization in the deep pelvis, while the ureteroneocystostomy itself was completed under direct vision through a lower transverse incision.

Postoperative adjuvant chemotherapy was selected according to pathologic stage, oncologic risk factors and prior neoadjuvant exposure. Case 1 (ypT3N0M0) received four cycles of CAPOX due to initial high-risk features (bowel obstruction and abscess formation). Cases 2 and 3 had stage III disease and received FOLFOX and CAPOX, respectively. Given the intensive preoperative chemotherapy, the duration of postoperative treatment was limited to four cycles of CAPOX or six cycles of FOLFOX to balance anticancer efficacy with cumulative toxicity. The optimal adjuvant regimen and duration after intensive neoadjuvant therapy for locally advanced CRC have not yet been firmly established, and further clinical data are needed.

Various options exist for urinary reconstruction after ureteral resection, including ureteroneocystostomy with or without a psoas hitch or Boari flap and urinary diversion using ileal conduits (5). In a systematic review and meta-analysis of urinary diversion after radical cystectomy (36,719 patients with an ileal conduit), the pooled incidence of urinary tract infection was 26.1% (1,048/4,013) and that of ureteric stricture was 7.0% (249/3,533) among the studies reporting these outcomes (10). In contrast, the psoas hitch procedure, originally developed and widely used in urology, allows ureteral reimplantation without the use of an intestinal segment in cases where the defect length and site are moderate. A recent multicenter series of 166 adults undergoing psoas hitch ureteroneocystostomy reported clinical failure in 8% of patients, with no significant deterioration in eGFR during follow-up (4). In line with the principle of avoiding intestinal use whenever possible in urinary reconstruction, our department prefers a psoas hitch ureteroneocystostomy when the tumor–ureter interface is near the common iliac artery, and a tension-free anastomosis can be achieved after bladder mobilization.

Given the high risk of recurrence, maintaining good renal function is crucial, as it can influence postoperative chemotherapy feasibility and significantly impact oncological prognosis. During follow-up, none of the patients showed clinically significant deterioration in renal function (serial serum creatinine values at 6 months, 1 year, 2 years, and at the latest follow-up are summarized in Table 2) or imaging findings suggestive of ureteral obstruction. In addition, no patient reported a subjective decline in urinary function-related quality of life.

This study has several limitations. It is a small, retrospective, single-institution case series without a control group, and urinary function was assessed by routine clinical follow-up rather than by validated patient-reported measures. The published series cited above differ in population, indication, and outcome definitions and are therefore not directly comparable. Our results should thus be interpreted as supporting the feasibility of psoas hitch ureteroneocystostomy in selected patients, not its superiority over other reconstructive strategies. Larger multicenter studies are warranted.

## Conclusion

NAC enabled downstaging and safe laparoscopic multivisceral resection in three patients with locally advanced colorectal cancer in whom ureteral invasion was suspected. When intraoperative findings suggest that safe dissection of the ureter from the tumor cannot be achieved, *en bloc* resection including the ureter should be performed. Psoas hitch ureteroneocystostomy was performed without major complications and with preserved renal function in all three patients. Although the present study is limited by its small sample size, our favorable perioperative and functional outcomes suggest that psoas hitch ureteroneocystostomy is a feasible organ-preserving option for selected patients with locally advanced colorectal cancer requiring ureteral resection.

## Data Availability

The original contributions presented in the study are included in the article/Supplementary Material, further inquiries can be directed to the corresponding author.
